# Men who have sex with men more often chose daily than event‐driven use of pre‐exposure prophylaxis: baseline analysis of a demonstration study in Amsterdam

**DOI:** 10.1002/jia2.25105

**Published:** 2018-03-30

**Authors:** Elske Hoornenborg, Roel CA Achterbergh, Maarten F Schim van der Loeff, Udi Davidovich, Jannie J van der Helm, Arjan Hogewoning, Yvonne THP van Duijnhoven, Gerard JB Sonder, Henry JC de Vries, Maria Prins, the Amsterdam PrEP Project team in the HIV Transmission Elimination AMsterdam Initiative

**Affiliations:** ^1^ Department of Infectious Diseases, Research and Prevention Public Health Service of Amsterdam Amsterdam the Netherlands; ^2^ Department of Infectious Diseases Public Health Service of Amsterdam STI Outpatient Clinic Amsterdam the Netherlands; ^3^ Department of Infectious Diseases Academic Medical Center Center for Immunity and Infection Amsterdam (CINIMA) University of Amsterdam Amsterdam the Netherlands; ^4^ Department of Dermatology Academic Medical Center University of Amsterdam Amsterdam the Netherlands; ^5^ Department of Infectious Diseases Public Health Service of Amsterdam Amsterdam the Netherlands; ^6^ Department of Infectious Diseases, Communicable Diseases Control Public Health Service of Amsterdam Amsterdam the Netherlands; ^7^ National Institute of Public Health and the Environment Center for Infectious Disease Control Bilthoven the Netherlands

**Keywords:** HIV infection, Prevention, pre‐exposure prophylaxis, PrEP, Men who have sex with men, transgender persons

## Abstract

**Introduction:**

The Amsterdam PrEP project is a prospective, open‐label demonstration study at a large sexually transmitted infection (STI) clinic. We examined the uptake of PrEP; the baseline characteristics of men who have sex with men (MSM) and transgender persons initiating PrEP; their choices of daily *versus* event‐driven PrEP and the determinants of these choices.

**Methods:**

From August 2015 through May 2016, enrolment took place at the STI clinic of the Public Health Service of Amsterdam, the Netherlands. MSM or transgender persons were eligible if they had at least one risk factor for HIV infection within the preceding six months. Participants were offered a choice between daily or event‐driven use of tenofovir/emtricitabine. Baseline data were analysed using descriptive statistics and multivariable analysis was employed to determine variables associated with daily *versus* event‐driven PrEP.

**Results:**

Online applications were submitted by 870 persons, of whom 587 were invited for a screening visit. Of them, 415 were screened for eligibility and 376 initiated PrEP. One quarter (103/376, 27%) chose event‐driven PrEP. Prevalence of bacterial STI was 19.0% and mean condomless anal sex (CAS) episodes in the preceding three months were 11. In multivariable analysis, older age (≥45 *vs*. ≤34, aOR 2.1, 95% CI 1.2 to 3.9), being involved in a steady relationship (aOR 1.7, 95% CI 1.0 to 2.7), no other daily medication use (aOR 0.6, 95% CI 0.3 to 0.9), and fewer episodes of CAS (per log increase aOR 0.7, 95% CI 0.6 to 0.9) were determinants for choosing event‐driven PrEP.

**Discussion:**

PrEP programmes are becoming one of the more important intervention strategies with the goal of reducing incident HIV‐infection and we were unable to accommodate many of the persons applying for this study. Offering a choice of dosing regimen to PrEP users may enable further personalization of HIV prevention strategies and enhance up‐take, adherence and cost‐effectiveness.

**Conclusions:**

The majority of participants preferred daily *versus* event‐driven use. Within this majority, a high number of CAS episodes before PrEP initiation was reported and we observed a high prevalence of STI. Determinants of choosing event‐driven PrEP were older age, fewer CAS episodes, no other daily medication use, and involved in a steady relationship.

## 1 Introduction

In the Netherlands, men who have sex with men (MSM) accounted for 65% of new HIV diagnoses in 2015 and HIV incidence has not been markedly declining 1. These findings indicate the urgent need for new methods of HIV prevention.

The use of daily oral tenofovir disoproxil fumarate combined with emtricitabine (TDF/FTC) as pre‐exposure prophylaxis (PrEP) effectively protects MSM against HIV infection 2. The Ipergay study showed comparable efficacy in MSM when using non‐daily event‐driven dosing of PrEP (i.e. taking PrEP before and after sexual contact) 3, 4.While daily PrEP use for at‐risk populations has been included in several guidelines (e.g. World Health Organization and U.S. Centers for Diseases Control and Prevention) 5, 6, event‐driven PrEP use has been recommended in French guidelines and the most recent European Aids Clinical Society guidelines 7, 8.

Issues that impede PrEP implementation in high‐income countries include uncertainty about the numbers and characteristics of PrEP users, as well as costs related to its use 9, 10. Event‐driven use could improve cost‐effectiveness since fewer pills are required 11, 12. For client‐centred and cost‐effective implementation, more information is needed on the uptake of both daily and event‐driven dosing regimens in real‐life settings and on the determinants of choosing between these regimens.

We started the Amsterdam PrEP (AMPrEP) demonstration project with the aim of assessing uptake of daily and event‐driven PrEP, at the participant's discretion, among HIV‐negative MSM and transgender persons at increased risk for HIV infection. This project was part of a comprehensive HIV‐reduction package offered at a large STI clinic. We assessed interest in PrEP use by reporting the number of study applicants during the study application period and evaluated baseline characteristics including STI prevalence and preference for daily *versus* event‐driven PrEP use. In addition, we examined the predominant self‐reported motives for PrEP use and choice of regimen, and analysed determinants for choosing event‐driven PrEP use.

## 2 Methods

### 2.1 Application procedures and study site

The AMPrEP project, a prospective, longitudinal, open‐label demonstration study, began in June 2015. A press release announced the start of the study and the number of available spots (n=370), followed by large‐scale media attention at the local and national level. We reached out to transgender people through their community organizations and via the centre for prostitution and health, which offers information, advice and support to sex workers including transgender persons. However, we did not launch a media campaign to recruit participants. The main inclusion criteria were published online and people interested in participating applied using a web‐based form. On this form, we asked them to select all applicable inclusion criteria. Since a high number of interested people was expected and we wanted to allow ample time for individuals to express their interest in participation, we decided not to include individuals on a first‐come, first‐serve basis, but instead opted for a four‐week application period (initial application period). All applicants then received a randomly‐assigned rank number for a subsequent screening visit. No stratified sampling of at‐risk subgroups (e.g. based on sexual behaviour characteristics, for young MSM, or transgender persons) was employed. During the first screening visit and prior to any assessments, applicants provided written informed consent to participate in the study. Visits took place from 3 August 2015 to 30 May 2016 at the STI outpatient clinic of the Public Health Service of Amsterdam (GGD). This is the largest STI clinic in the Netherlands, performing about 45,000 consultations yearly; 38% of clients are MSM. In 2017, we diagnosed 92 HIV infections, 77 of these were MSM.

The study was approved by the ethics board of the Academic Medical Center, Amsterdam, the Netherlands (NL49504.018.14). The AMPrEP project is part of the HIV Transmission Elimination AMsterdam (H‐TEAM) initiative, a multidisciplinary and integrative approach to stop the urban epidemic (Hteam.nl). The AMPrEP study was registered at the online Dutch trial registry (registration number NTR5411) and the protocol is available online 13.

### 2.2 Eligibility criteria

HIV‐negative MSM and transgender persons (male‐to‐female and female‐to‐male) who have sex with men were eligible if they were at least 18 years of age and had one or more of the following risk factors for HIV infection within six months prior to the screening visit: condomless anal sex (CAS) with casual partners, at least one bacterial STI (i.e. syphilis, rectal or urethral chlamydia or gonorrhoea), use of post‐exposure prophylaxis (PEP) after a sexual risk incident, or an HIV‐positive sexual partner with a detectable viral load. We excluded people if they tested positive for hepatitis B surface antigen (HBsAg); had creatinine clearance rate according to the Cockroft‐Gault equation less than 60 mL/minute 14; had more than trace protein in urine, or had other contra‐indications for TDF/FTC use (see protocol 13). In case of symptoms or signs suggestive of acute HIV infection, we performed rapid point‐of‐care HIV RNA testing.

### 2.3 PrEP dosing regimen

Participants were proposed a choice of free‐of‐charge daily or event‐driven PrEP (TDF/FTC) use. Also, no costs were incurred on the participants for renal function and STI testing. Daily use consisted of one pill every day and event‐driven use two tablets taken between 24 hours and 2 hours before CAS, followed by one tablet every 24 hours up to 48 hours after the last episode of sexual intercourse 3. In the decision‐making process, we used shared and informed decision making, (i.e. we explained both PrEP regimens and answered questions), thus providing the necessary information for participants to select the option most‐adapted to their lifestyle. No advice was given on the suitability of their decision of PrEP regimen. In addition, we informed participants that switching PrEP regimen was allowed at each subsequent study visit.

### 2.4 Study visits

**Figure 1:**
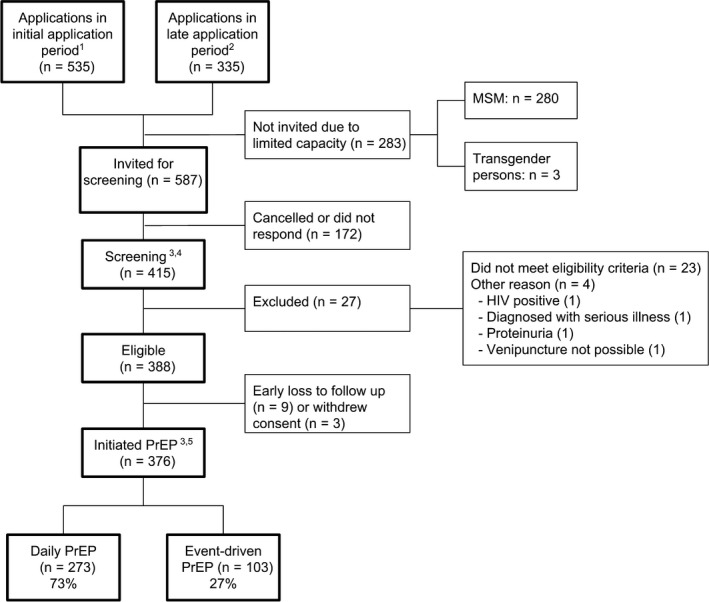
Overview of the numbers of applications, people screened and starting daily and event‐driven PrEP in AMPrEP. Baseline characteristics were obtained at the screening or PrEP initiation visit. (1) From 22 June to 20 July 2015. (2) From 20 July 2015 to 12 April 2016. (3) Screening and PrEP initiation took place from August 2015 until June 2016. (4) At the screening visit, we obtained informed consent and took samples to test for sexually transmitted infections, including HIV and hepatitis B virus infection, and kidney function. (5) At the PrEP initiation visit, we took samples for pooled HIV, hepatitis B and hepatitis C virus testing; we performed a semi‐structured interview on participants’ main reason to start PrEP and for choosing daily or event‐based PrEP. We also collected self‐reported questionnaire data.

At the screening visit, eligibility criteria were verified. Urine, blood and pharyngeal/anal samples were taken to test for STI, hepatitis B virus (HBV), HIV, serum creatinine and urine protein (Figure 1). One to four weeks later at PrEP initiation, we verified that all diagnosed STI had been treated according to clinic protocols, and if not we provided immediate treatment; eligibility criteria were confirmed, a self‐administered questionnaire was completed, and blood was taken to test for HIV, hepatitis C virus (HCV) and HBV infections. Participants were asked to return for follow‐up visits one month after the PrEP initiation visit and every three months thereafter. They will be provided with PrEP until June 2018.

### 2.5 Diagnostic testing

We performed HIV and STI testing according to routine STI clinic protocols. This included HIV antigen and antibody testing (LIAISON XL Murex HIV Ag/Ab, Diasorin, Saluggia, Italy) with immunoblot confirmation (INNO_LIPA HIV I/II Score, Fujirebio, Ghent, Belgium), and serology testing for syphilis (LIAISON Treponema Screen, Diasorin). If indicated, direct microscopy (dark‐field or Gram‐stained smear) was performed for samples taken from rectal or urethral ulcers or discharge to detect the presence of *Neisseria gonorrhoeae* or *Treponema pallidum*. Nucleic amplification testing for *Chlamydia trachomatis* and *Neisseria gonorrhoeae* (Aptima combo 2, Hologic Gen‐Probe Inc., San Diego, USA) was performed on urine, anal and pharyngeal swabs. Samples from participants without a history of HBV vaccination were screened for anti‐Hepatitis B core and anti‐Hepatitis B surface antibodies (LIAISON Anti‐HBc and anti‐HBs II, Diasorin) and, if negative, participants were offered HBV vaccination.

In addition to the routine STI clinic protocol, blood taken at the PrEP initiation visit was tested for anti‐HCV antibodies (Architect anti‐HCV, Abbott Laboratories, Wiesbaden, Germany) with immunoblot confirmation (INNO‐LIPA HCV Score, Fujirebio, Ghent, Belgium) as was previously described 15. Participants were also tested for the presence of HIV RNA, HCV RNA and HBV DNA in pools of six plasma samples (COBAS Taqscreen MPX Test v2.0, Roche Diagnostics, Mannheim, Germany). Samples in HBV DNA‐positive pools were tested for HBsAg (Architect HBsAg, Abbott Laboratories, Wiesbaden, Germany), and HBsAg‐positive samples were individually tested for HBV DNA (CAP‐CTM HBV test v2.0, Roche Diagnostics, Mannheim, Germany). None of the pools tested positive for HIV‐1/2 RNA.

### 2.6 Measurements

#### 2.6.1 Participant characteristics and risk behaviour

At the PrEP initiation visit, participants completed a self‐administered questionnaire on risk behaviour over the past three months, socio‐demographic characteristics (e.g. postal code, sexual preference, education, employment, income and living situation), and their main reason for participating in the AMPrEP project (from a list of 10 predefined options). Questions were asked on concern of acquiring HIV using a Likert scale from one (very concerned) to seven (not concerned at all). Educational level was divided into low (primary school and lower secondary vocational education), medium (intermediate and higher secondary general education, senior secondary vocational and pre‐university education) or high (higher professional or university education). Regarding sexual behaviour, participants were asked about number and type (steady, known casual, or anonymous) of sexual partners, the number and type (insertive or receptive) of anal sex episodes, and condom use. Using lay terms, questions were asked on substance use, both in general and around the time of sex, and whether they had injected any drugs. Chemsex was defined as using gamma‐hydroxybutyrate (GHB)/gamma‐butyrolactone (GBL), mephedrone or crystallized methamphetamine around the time of sex 16. Finally, participants were asked if they routinely used medication and pharmaceutical interactions with TDF/FTC were checked.

#### 2.6.2 Other measurements at PrEP initiation

Participants were asked the most important reason to participate in the AMPrEP project and to complete the Mental Health Inventory screening test (MHI‐5), Alcohol Use Disorders Identification Test (AUDIT), Drug Use Disorders Identification Test (DUDIT), and sexual compulsivity scale (SCS) 17. The following score cutoffs were used to identify specific problems: 60 for MHI‐5 (i.e. lower than 60 suggests an anxiety or depressive mood disorder) 18, 19, eight for AUDIT (i.e. eight and higher suggests harmful alcohol use) 20, eight for DUDIT (i.e. eight and higher suggests drug‐related problems) 21 and 24 for SCS (i.e. 24 and higher suggests a greater impact of sexual thoughts on daily functioning and an inability to control sexual thoughts or behaviours) 22, 23.

Semi‐structured interviews were used to identify the two most important motives for choosing daily *versus* event‐driven PrEP at the PrEP initiation visit.

### 2.7 Statistical analyses

First, we described the number of applicants, number screened and number initiating PrEP. Second, we reported the socio‐demographic characteristics, risk behaviour, STI diagnoses, choice of PrEP dosing regimen and motives for participation and choice. Third, we compared characteristics between participants who chose daily PrEP compared to event‐driven PrEP using Chi‐squared tests for categorical data and student t‐test or rank sum tests for continuous data. Fourth, variables associated at *p *< 0.15 in univariable analysis were included in a multivariable logistic regression model while forcing inclusion of age as a covariable. In case of collinearity between variables (e.g. number of anal sex partners and number of anal sex episodes), only one of the collinear variables was included. Number of CAS episodes was natural log transformed. Using a backward step‐wise procedure, we retained variables with a *p* < 0.05 (according to the likelihood ratio test) in the final multivariable model. All analyses were performed using STATA Intercooled 13.1 (STATA Corporation, College Station, TX, USA).

## 3 Results

### 3.1 Individuals applying and assessed for participation

In total, 870 online applications were submitted from June 2015 to April 2016: 535 during the 4‐week initial application period and 335 in the nine months thereafter (Figure 1). Between August 2015 and June 2016, 587 of 870 persons were invited for a screening visit, of whom 415 (77.6%) attended their visit. Of them, 376 (90.6%) initiated PrEP. Twenty‐three people did not meet any of the four risk factors for HIV infection (see methods), and exclusion criteria disqualified four people from inclusion (Figure 1).

### 3.2 Socio‐demographic characteristics and STI prevalence

Two of the 376 participants self‐identified as male‐to‐female transgender persons and 374 as MSM (Table 1). Most of the participants were white (85.1%) and had a high educational level (76.1%). The median age was 39 years [interquartile range (IQR), 32 to 48].

**Table 1:** Baseline characteristics of daily and event‐driven PrEP users in AMPrEP 2015 to 2016

	Total (N=376)		Event driven PrEP users (N=103)		Daily PrEP users (N=273)		*p* value
	N	%^a^	n	%^a^	n	%^a^	
Demographic characteristics							
Age (years)							
Median [IQR]	39	[32 to 48]	44	[35 to 51]	38	[30 to 47]	<0.001
≤34	126	33.5	25	24.3	101	36.9	0.016
35 to 44	112	29.8	29	28.2	83	30.4	
≥45	138	36.7	49	47.6	89	32.6	
Gender identity							0.473
Male	374	99.5	102	99.0	272	99.6	
Transgender woman	2	0.5	1	1.0	1	0.4	
Self‐declared ethnicity^b^							0.160
White	315	85.1	92	89.3	223	83.5	
Non‐white	55	14.9	11	10.7	44	16.5	
Country of origin							0.811
Netherlands	295	78.5	82	79.6	213	78.0	
Other high‐income country	39	10.4	9	8.7	30	11.0	
Other	42	11.2	12	11.7	30	11.0	
Residence							0.155
Amsterdam	230	61.2	69	67.0	161	59.0	
Other	146	38.8	34	33.0	112	41.0	
Educational level							0.071
Low and middle	90	23.9	18	17.5	72	26.4	
High	286	76.1	85	82.5	201	73.6	
Employment^d^							0.071
Yes	290	77.1	80	77.7	210	78.1	
Student	31	8.2	3	2.9	28	10.4	
No, volunteer	7	1.9	3	2.9	4	1.5	
No, unable due to disability	6	1.6	1	1.0	5	1.9	
No, retired	19	5.1	8	7.8	11	4.1	
No, unemployed	19	5.1	8	7.8	11	4.1	
Monthly net income level^e^							0.423
Low (≤€1700)	99	27.7	25	25.0	74	28.8	
Middle (€1701 to €2950)	151	42.3	40	40.0	111	43.2	
High (>€2950)	107	30.0	35	35.0	72	28.0	
Current steady relationship^d^							0.060
No	208	55.9	49	48.0	159	58.9	
Yes	164	44.1	53	52.0	111	41.1	
Living situation							0.029
Alone	200	53.9	61	59.2	139	50.9	
With partner	121	32.2	35	34.0	86	31.5	
With others	55	14.6	7	6.8	48	17.6	
Sexual behaviour characteristics							
Sexual preference^c^							0.890
Exclusively homosexual	296	78.7	81	79.4	215	78.8	
Not exclusively homosexual	79	21.0	21	20.6	58	21.3	
Concerned about acquiring HIV (scale 1–7)^f^							
Median [IQR]	3	[2 to 4]	3	[2 to 4]	3	[2 to 4]	0.843
Eligibility criteria^g^							
Sexually transmitted infection^h^	135	35.9	29	28.2	106	38.8	0.054
Post‐exposure prophylaxis used	27	7.2	4	3.9	23	8.4	0.128
CAS with casual partner(s)	359	95.5	100	97.1	259	94.9	0.356
HIV‐positive partner with a detectable viral load	9	2.4	1	1.0	8	2.9	0.268
Number of eligibility criteria							0.037
1	235	62.5	75	72.8	159	58.5	
2	127	33.8	25	24.3	102	37.5	
3	14	3.7	3	2.9	11	4.0	
STI diagnosed at PrEP initiation							
Anorectal chlamydia^i^	19	5.1	4	3.9	15	5.5	0.516
Chlamydia^i^ (any site)	32	8.6	6	5.8	26	9.6	0.244
Anorectal gonorrhoea^i^	25	6.7	7	6.8	18	6.6	0.958
Gonorrhoea^i^ (any site)	36	9.6	11	10.7	25	9.2	0.670
Syphilis (stage 1 or 2)^i^	6	1.6	1	1.0	5	1.9	0.548
Hepatitis C antibody or RNA^c^	18	4.8	4	3.9	14	5.2	0.609
Hepatitis B surface antigen	2	0.5	0		2	0.7	
Anal chlamydia or gonorrhoea^i^	42	11.2	11	10.7	31	11.4	0.835
Any bacterial STI^i^^,^ j (any site)	71	19.0	18	17.5	53	19.6	0.647
STI diagnosed at clinic preceding PrEP initiation							
Any bacterial STI preceding year^j^^,^ k (any site)	103	45.2	23	36.0	80	48.8	0.096
Any bacterial STI preceding three years^j^^,^ l (any site)	155	61.5	41	58.6	114	62.6	0.586
Number of anal sex partners (3M)							
Median [IQR]	15	[6 to 30]	10	[5 to 25]	15	[8 to 30]	0.015
0 to 1	8	2.1	4	3.9	4	1.5	0.116
2 to 5	67	17.8	23	22.3	44	16.1	
6 to 9	52	13.8	16	15.5	36	13.2	
10 to 24	116	30.9	33	32.0	83	30.4	
≥25	133	35.4	27	26.2	106	38.8	
Number of condomless anal sex episodes (3M)							
Median [IQR]	11	[4 to 23]	8	[2 to 17]	12	[5 to 25]	0.008
0 to 1	51	13.6	22	21.4	29	10.6	0.061
2 to 5	73	19.4	20	19.4	53	19.4	
6 to 9	49	13.0	15	14.6	34	12.5	
10 to 24	115	30.6	27	26.2	88	32.2	
≥25	88	23.4	19	18.5	69	25.3	
Position during condomless anal sex (3M)							0.053
None	29	7.7	14	13.6	15	5.5	
Insertive only	65	17.3	16	15.5	49	18.0	
Receptive only	63	16.8	19	18.5	44	16.1	
Both insertive and receptive	219	58.2	54	52.4	165	60.4	
Most high‐risk type partner (3M)^c^							0.238
Anonymous partner	344	92.0	89	88.1	255	93.4	
Known casual/ex steady partner	27	7.2	11	10.9	16	5.9	
Steady partner	3	0.8	1	1.0	2	0.7	
Mental health characteristics and drug use							
Depressive or anxiety symptoms							0.641
MHI‐5 score <60^m^ (symptoms)	78	20.7	23	22.3	55	20.2	
MHI‐5 score ≥60^n^ (no symptoms)	298	79.3	80	77.7	218	79.9	
Alcohol use disorder identification test (AUDIT)^o^							0.878
Score <8^p^ (no indication)	267	72.0	74	72.6	193	71.8	
Score ≥8^q^ (indication)	104	28.0	28	27.5	76	28.3	
Drug use disorders identification test (DUDIT)^r^							0.119
Score <8^s^ (no indication)	236	64.3	71	69.6	165	60.9	
Score ≥8^t^ (indication)	137	35.7	31	30.4	106	39.1	
Sexual compulsivity scale^c^							0.394
Score <24^u^ (no indication)	291	77.6	83	80.6	208	76.5	
Score ≥24^v^ (indication)	84	22.4	20	19.4	64	23.5	
Alcohol use during sex (3M)^w^	235	63.3	62	61.4	173	64.1	0.632
Drug use during sex (3M)							
Amphetamine^w^	68	18.3	18	17.8	50	18.5	0.877
Cannabis^x^	104	28.2	26	25.7	78	29.1	0.522
Cocaine^b^	98	26.5	27	26.7	71	26.4	0.948
Erectile dysfunction drugs^b^	260	70.5	67	66.3	193	72.0	0.286
GHB/GBL^w^	146	39.4	39	38.6	107	39.6	0.859
Ketamine^x^	66	17.9	18	18.0	48	17.8	0.972
Methamphetamine^w^	34	9.2	7	7.0	27	10.0	0.380
Mephedrone^w^	31	8.2	11	11.0	20	7.4	0.264
Nitrites^w^	259	69.8	66	65.4	193	71.5	0.252
XTC/MDMA^b^	161	43.5	40	40	121	44.8	0.407
Other^y^	36	10.1	10	10.4	26	10.0	0.908
Any drugs during sex (3M)^z^	330	89.7	89	89.0	241	89.9	0.795
Chemsex (3M)*	156	42.0	44	43.6	112	41.5	0.718
Injecting drug use (3M)^w^	15	4.0	3	3.0	12	4.4	0.521
Daily medicine use	114	30.3	25	24.3	89	32.6	0.117

AMPrEP, Amsterdam PrEP project; PrEP, pre‐exposure prophylaxis; IQR, interquartile range; STI, sexually transmitted infection; MHI5, Mental Health Inventory 5; RNA, Ribonucleic acid; CAS, condomless anal sex; GHB, γ‐hydroxybutyrate; GBL, γ‐Butyrolactone; XTC, ecstasy; MDMA, 3,4‐Methylenedioxymethamphetamine.

^a^Percentages may not total 100 because of rounding, percentage is within group of PrEP users; ^b^6 missing; ^c^1 missing; ^d^4 missing; ^e^19 missing/did not want to say; ^f^1 very concerned, 7 not concerned at all; ^g^In the previous 6 months, self reported risk factors; ^h^STI include only rectal, urethral chlamydia/gonorrhea or syphilis; ^i^2 missing; ^j^Chlamydia, gonorrhoea or syphilis diagnosed at STI clinic Amsterdam; ^k^148 missing; ^l^124 missing; ^m^No indication of an anxiety or depressive mood disorder; ^n^Possible indication of an anxiety or depressive mood disorder; ^o^5 missing; ^p^1 No indication of alcohol use disorder; ^q^Indication of possible alcohol use disorder; ^r^3 missing; ^s^No indication of drug use disorder; ^t^Indication of possible drug use disorder; ^u^No indication of sexual compulsivity; ^v^Indication of possible sexual compulsivity; ^w^5 missing; ^x^7 missing; ^y^20 missing; others include: opiates (8), mushrooms (1), methylphenidate (8), testosterone (7), Methoxetamine (MXE, 2), 4‐broom‐2,5‐dimethoxyfenethylamine (2CB, 5), Lysergic acid diethylamide (LSD, 3), 4‐Fluoroamphetamine (4FA, 15), 4‐Methylethcathinone (4‐MEC, 1); ^z^other than alcohol, 8 missing; *defined as the use of GHB/GBL or Methamphetamine or Mephedrone during sex; 3M: in the previous 3 months.

At PrEP initiation, 71 of 376 (19.0%) were diagnosed with a bacterial STI. Overall, 42 (11.2%) cases of anal gonorrhoea or chlamydia and 6 (1.6%) cases of syphilis were diagnosed. Moreover, 18 (4.8%) were positive for anti‐HCV (n=17) and/or HCV RNA (n=15). Of 228 participants who had tested at the STI clinic within the preceding year, 103 (45.2%) had been diagnosed with a bacterial STI. Two people initiating daily PrEP tested HBsAg‐positive, but were enrolled despite this exclusion criterion. One had been using event‐driven PrEP, unmonitored and obtained elsewhere, while the second was mistakenly included. We referred both to specialist care to have their HBV infection monitored. Furthermore, we allowed continuation of study participation and advised to use PrEP daily to prevent hepatic flares when PrEP is interrupted.

### 3.3 Sexual risk behaviour, drug use and other measurements

Characteristics of risk behaviour and drug use in the three months prior to PrEP initiation are described in Table 1. The median number of anal sex partners in the three months before PrEP initiation was 15 [IQR 6 to 30] and the median number of CAS episodes was 11 (IQR 4 to 23). Of all participants, 282 (75.0%) reported receptive CAS, and 203 (54.0%) reported at least ten CAS episodes in the preceding three months.

Of all participants, 156 (42.0%) reported chemsex and 15 (4.0%) reported injecting drug use in the preceding three months. More than one third (35.7%) scored eight or higher on DUDIT and 28.0% scored eight or higher on AUDIT, suggesting drug and alcohol use disorder respectively. According to the MHI‐5, 20.7% scored below 60, suggesting a depressive mood and/or anxiety disorder.

### 3.4 Preferences for daily *versus* event‐driven PrEP use

Of 376 persons who initiated PrEP, 273 (72.6%) opted for daily use. Median age of daily PrEP users was 38 years [IQR 30 to 47], 111 (41.1%) were involved in a steady relationship and 86 (31.5%) were living with their partner. Daily PrEP users reported a median number of 15 [IQR 8 to 30] anal sex partners in the preceding three months.

Those who opted for event‐driven PrEP had a median age of 44 years [IQR 35 to 51], 53 (52.0%) were involved in a steady relationship and 35 (34.0%) were living with their partner. In the preceding three months, they reported a median number of 10 [IQR 5 to 25] anal sex partners.

### 3.5 Motives for participation and choice of PrEP regimen

**Figure 2:**
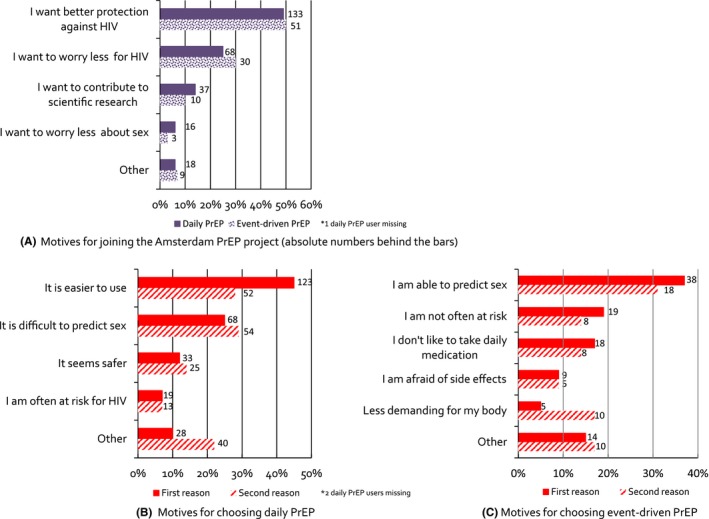
**(A)** Main motives for joining the Amsterdam PrEP project (one motive per participant); **(B)**. Main motives (two per participant) for choosing daily PrEP;** (C)**. Main motives (two per participant) for choosing event‐driven PrEP. Proportions of all participants who reported a first/second reason on the *x*‐axis and absolute numbers behind the bars.

The main reasons for participation were to have better protection against HIV (*n*=184, 49.1%) or to worry less about contracting HIV (*n*=98, 26.1%) (Figure 2A). The two main reasons for PrEP regimen choice were daily *PrEP* – “it is easier to use” and “it is difficult to predict when I will have sex;” event‐driven *PrEP* – “I am able to predict sex” and “I am not often at risk” (Figure 2B and C).

### 3.6 Determinants for choosing event‐driven PrEP use

In univariable analyses, older age was positively associated with choosing event‐driven PrEP, whereas persons with higher numbers of anal sex partners and CAS episodes and more eligibility criteria were less likely to choose event‐driven PrEP (Tables 1 and 2). Several other factors, including educational level and a higher score on the screening tool for drug disorder were not associated with choice of PrEP regimen. In multivariable analyses, older age (≥45 *vs*.≤34, adjusted odds ratio (aOR) 2.1, 95% CI 1.2 to 3.9) and being involved in a steady relationship (aOR 1.7, 95% CI 1.0 to 2.7) were positively associated with event‐driven PrEP; daily medication use (aOR 0.6, 95% CI 0.3 to 0.9) and more CAS episodes in the preceding three months (per log increase aOR 0.7, 95% CI 0.6 to 0.9) were negatively associated with event‐driven PrEP.

**Table 2:** Univariable and multivariable analysis of determinants for choosing event‐driven PrEP among 376 participants in AMPrEP 2015 to 2016

	n choosing event‐driven/total^a^ (%)	Univariable OR [95% CI]	Multivariable^b^ OR [95% CI]	*p* value
Age (years)				0.027
≤34	25/126 (19.8)	1	1	
35 to 44	29/112 (25.9)	1.4 [0.8 to 2.6]	1.3 [0.7 to 2.4]	
≥45	49/138 (35.5)	2.2 [1.3 to 3.9]	2.1 [1.2 to 3.9]	
Educational level				
Low and middle	18/90 (20.0)	1		
High	85/286 (29.7)	1.7 [1.0 to 3.0]		
Employment/student^c^				
Yes	83/238 (25.9)	0.5 [0.3 to 1.0]		
No	20/31 (39.2)			
Current steady relationship^c^				0.045
No	49/208 (23.6)	1	1	
Yes	53/164 (32.3)	1.5 [1.0 to 2.4]	1.7 [1.0 to 2.7]	
Living situation^d^				
Alone	61/200 (30.5)	1		
With partner	35/121 (28.9)	0.9 [0.6 to 1.5]		
With others	7/55 (12.7)	0.3 [0.1 to 0.8]		
Number of eligibility criteria^e^				
1	75/234 (31.9)	1		
2 or 3	28/141 (19.9)	0.5 [0.3 to 0.9]		
Ln of number of anal sex partners^d^				
Per 1 increase		0.8 [0.6 to 1.0]		
Ln of number of condomless anal sex episodes				0.005
Per 1 increase		0.8 [0.6 to 1.0]	0.7 [0.6 to 0.9]	
Position during condomless anal sex				
None	14/29 (48.3)	1		
Top only	16/65 (24.6)	0.3 [0.1 to 0.9]		
Bottom only	19/63 (30.2)	0.5 [0.2 to 1.1]		
Versatile	54/219 (24.7)	0.4 [0.2 to 0.8]		
Drug use disorder identification test (DUDIT)^f^				
Score <8 (no indication)	71/236 (30.8)	1		
Score ≥8 (indication)	31/137 (22.6)	0.7 [0.4 to 1.1]		
Daily medicine use				0.041
No other daily medicine use	78/262 (29.8)	1	1	
Other daily medicine use	25/114 (21.9)	0.7 [0.4 to 1.1]	0.6 [0.3 to 0.9]	

PrEP, pre‐exposure prophylaxis; AMPrEP, Amsterdam PrEP project; DUDIT, Drug Use Disorders Identification Test.

^a^Percentages may not total 100 because of rounding, percentage is within variable group; ^b^multivariable model is based on 372 participants; ^c^4 missing; ^d^not included in multivariable model due to collinearity; ^e^1 missing; ^f^2 missing.

## 4 Discussion

In the Amsterdam PrEP project, the demand for PrEP among MSM largely exceeded capacity. The majority of the participants in this Dutch demonstration project preferred daily *versus* event‐driven use. Within this majority, a high number of CAS episodes before PrEP initiation was reported and a high prevalence of STI, including HCV was observed. Furthermore, 42% reported engaging in chemsex. Determinants of choosing event‐driven PrEP were older age, fewer CAS episodes in the preceding three months, no other daily medication use, and being in a steady relationship.

To the best of our knowledge, we are the first to report on the preferences between daily and event‐driven PrEP among MSM and the reasons for such preferences. Although most people opted for daily use, about one quarter preferred event‐driven use. People who opted for event‐driven PrEP reported fewer CAS episodes and thus would require PrEP less often for protection; they were less likely to take daily medication whereby daily PrEP could be conveniently integrated. Providing individuals a choice between daily or event‐driven PrEP would appear to enable further personalization of their HIV prevention strategy and could accompany increased adherence and resulting protection. In addition, as event‐driven users take fewer PrEP pills, offering this choice has the potential to reduce cost at the individual and population level alike, and improve cost‐effectiveness 11, 12. In a PrEP rollout study in France, uptake of daily PrEP is lower than in our project: 41% of individuals preferred daily PrEP and 59% event‐driven PrEP 4. The difference in regimen preference between the French study and our own might reflect the fact that French providers and MSM were more familiar with event‐driven dosing from the randomized‐controlled trial Ipergay in France 3.

The demand for PrEP was reflected by the large number of submitted applications to participate in AMPrEP over a short time period. Nevertheless, we clearly stated to participants that late applicants were unlikely to be enrolled due to limited capacity. Potential applicants interested in PrEP could have perceived their chance of inclusion as low and thus were discouraged from participating, implying that the number of those actually interested in initiating PrEP in Amsterdam are higher than the number of applicants to the AMPrEP project. Currently, few data on intention to use PrEP in the Netherlands have been published 24, 25 and only crude estimates of the number of MSM willing to take PrEP are available 26, 27.

The participants initiating PrEP in this study were indeed at substantial risk for HIV infection, as shown by a high median number of CAS episodes and high STI prevalence. The proportion with a bacterial STI (19.0%) at PrEP initiation corresponded to other PrEP cohorts (15.9% to 28.8%) 3, 28. However, the prevalence of HCV infection was higher in our study 3, 15, 29, indicating that STI testing strategies should include HCV among HIV‐negative MSM initiating PrEP.

The proportion of men reporting chemsex in the three months before PrEP initiation is high and in line with two other European PrEP cohorts 3, 19 and with studies in the larger MSM community 30, 31. Moreover, in our study, a large proportion scored low on MHI‐5 or high on the DUDIT, AUDIT, or SCS screening tools, indicating risk for anxiety or depressive mood disorder, alcohol and drug use disorder, or sexual compulsivity. Even though sexual minorities are known to have more mental health problems (e.g. alcohol dependence 5.4% in heterosexuals compared with 10.4% in non‐heterosexuals) 32, these scores are alarmingly high. Addressing and, if indicated, referral for substance use, sexual compulsivity and mental health should be integrated into PrEP programmes, and the quarterly visits offer ample opportunity to do so.

Despite efforts to inform all PrEP‐eligible populations, our study included very few transgender persons, as was the case in other PrEP studies from Europe and elsewhere 3, 19, 33, 34. To increase their inclusion, future studies will require additional and specifically‐tailored PrEP campaigns and programmes based on research identifying the specific needs of transgender people at risk for HIV infection (e.g. providing PrEP at gender‐affirming care facilities) 35, 36.

One limitation of our study is that, despite the project being conducted in an STI clinic offering routine sexual healthcare, choices for daily and event‐driven PrEP may not reflect those in the population at large. It should be mentioned that PrEP was offered free‐of‐charge, which is currently not the case in most countries. More people might opt for event‐driven PrEP under the premise that future practice would require payment. Furthermore, our project is the first to offer access to a free‐of‐charge PrEP programme in the Netherlands and as a result, we may have attracted a higher number of early adopters who may not be representative of the wider MSM community.

The major strength of this study is the knowledge generated on PrEP use and choice of dosing regimens in a real‐world setting. So far, little data have been available, especially in Europe. The STI clinic in Amsterdam represents an ideal location for conducting this PrEP project given the frequent previous STI testing among participants (Table 1). Our initially high number of online applications provides further support for implementation of PrEP at STI clinics.

In conclusion, we were unable to accommodate many of the persons applying for this study in the Netherlands. Participants were willing to start daily or event‐driven PrEP as part of their routine STI care. They were able to choose a dosing regimen and the larger preference towards daily use was probably related to their risk behaviour. Offering a choice of dosing regimen may enhance uptake, adherence, and cost‐effectiveness. The results of this study are important for policy makers, health insurers, and healthcare professionals concerned with implementation of efficient and cost‐effective PrEP programmes in high‐income countries. Until the availability of new interventions, such as an HIV vaccine, PrEP programmes are becoming one of the more important intervention strategies with the goal of reducing incident HIV infection. Therefore, it is of importance to provide the option between daily and event‐driven PrEP for all people at high risk of acquiring HIV.

## Competing interests

We received the study drug for the Amsterdam PrEP study from Gilead Sciences. EH: my institute received financial reimbursement for time I spent serving on advisory boards of Gilead Sciences, and a speaker fee from Janssen‐Cilag.

## Authors’ contributions

EH, RA, MP, HdV, MSvdL, UD, GS, AH and JvdH contributed to study concept and design. EH, RA, MP, HdV, MSvdL, UD and JvdH contributed to acquisition, analysis or interpretation of the data. EH and RA contributed to drafting of the manuscript. EH, RA, MP, HdV, UD, GS, JvdH, MSvdL, AH and YvD contributed to critical revision of the manuscript. RA, MSvdL and JvdH contributed to statistical analysis. EH, MP, MSvdL and YvD obtained funding.

## Supporting information


**Annex S1.** Acknowledgements of the H‐TEAM consortium.
