# Supplementary material for: Men who have sex with men more often chose daily than event‐driven use of pre‐exposure prophylaxis: baseline analysis of a demonstration study in Amsterdam
Source: J Int AIDS Soc. 2018 Mar 30;21(3):e25105. doi: 10.1002/jia2.25105 (PMC5878413; doi:10.1002/jia2.25105)
Supplement: Supplementary file 1 — Annex S1. Acknowledgements of the H‐TEAM consortium. [file JIA2-21-e25105-s001.docx]

**Annex 1**: Acknowledgements of the H-TEAM consortium

H-TEAM initiative study group members:

H-TEAM Steering Committee: J.E.A.M. van Bergen^6;4;5;30^, G.J. de Bree^1;2^, P. Brokx^8^, F. Deug^6^, M. Heidenrijk^1^, M. Prins ^2;3^, P. Reiss^1;7^ (chair), M. van der Valk^2^

H-TEAM Core Project Group**:** J.E.A.M. van Bergen^6;4;5;30^, G.J. de Bree^1;2^ (chair), P. Brokx^8^, U. Davidovich^3^, S.E. Geerlings^2^, E. Hoornenborg^3^, A. Oomen^6^, A. van Sighem^7^, W. Zuilhof^6^

H-TEAM Project Management: M.L. Groot Bruinderink^1^, W. Zikkenheiner^1^

H-TEAM additional collaborators:

R.C.A. Achterbergh^3^, M. van Agtmael^24^, J. Ananworanich^22^, D. Van de Beek^17^, G.E.L. van den Berk^11^, D. Bezemer^7^, A. van Bijnen^6^, W.L. Blok^11^, M. Bomers^24^, C.A.B. Boucher^13^, W. Brokking^26^, D. Burger^20^, K. Brinkman^11^, N. Brinkman^33^, M. de Bruin^12^, S. Bruisten^3^, R. van Crevel^29^, L. Dellemann^6^, M. Dijkstra^3^, Y.T. van Duijnhoven^3^, A. van Eeden^26^, L. Elsenburg^26^, C. Ester^7^, P.H.J. Frissen^11^, T.B.H. Geijtenbeek^18^, M.H. Godfried^2^, J. van Gool^3^, A. Goorhuis^2^, M. Groot^26^, C.A. Hankins^1^, A. Heijnen^31;32^, M.M.J Hillebregt^7^, A. Hogewoning^3^, J.W. Hovius^2^, K. de Jong^3^, R. Kemp^33^, N.A. Kootstra^19^, R.A. Koup^21^, M. Kroone^3^, F.P. Kroon^16^, F. Lauw^25^, K. Lettinga^27^, I. Linde^3^, L. May^3^, E. Meddens^6^, J.T. van der Meer^2^, S. van Meeteren^3^, T. Mouhebati^6^, J. Mulder^25^, F.J. Nellen^2^, A. Nijsters^6^, H. Nobel^2^, P. Oostvogel^3^, E.L.M. Op de Coul^5^, E. Peters^24^, I.S. Peters^3^, T. van der Poll^2^, O. Ratmann^28^, C. Rokx^14^, M.S. van Rooijen^3^, M.F. Schim van der Loeff^2,3;10^, W.E.M. Schoute^11^, G.J. Sonder^3^, J. Veenstra^27^, A. Verbon^14^, F. Verdult^8^, G.R. Visser^7^, J. de Vocht^24^, H.J. de Vries^3;9;10^, S. Vrouenraets^25^, M. van Vugt^2^, W.J. Wiersinga^2^, F.W. Wit^2^, L.R. Woittiez^2^, S. Zaheri^7^, P. Zantkuijl^6^, M.C. van Zelm^23^, F.R. Zuure^3^.

^1^ Department of Global Health, Academic Medical Center, and Amsterdam Institute for Global Health and Development, Amsterdam, the Netherlands

^2^ Department of Internal Medicine, Division of Infectious Diseases, Academic Medical Center Amsterdam, the Netherlands

^3^ Department of Infectious Diseases, Public Health Service of Amsterdam, Amsterdam, the Netherlands

^4^ Department of General Practice, Academic Medical Center, University of Amsterdam, the Netherlands

^5^ Epidemiology and Surveillance Unit, Centre for Infectious Disease Control, National Institute of Public Health and the Environment, the Netherlands

^6^ STI AIDS Netherlands, Amsterdam, the Netherlands

^7^ Stichting HIV Monitoring, Amsterdam, the Netherlands

^8^ Dutch Association of PLHIV, Amsterdam, the Netherlands

^9^ Department of Dermatology, Academic Medical Center (AMC), University of Amsterdam, Amsterdam, the Netherlands

^10^ Center for Infection and Immunology, Amsterdam (CINIMA), Academic Medical Center (AMC), University of Amsterdam, Amsterdam, the Netherlands

^11^ Department of internal medicine, Onze Lieve Vrouwe Gasthuis, Amsterdam, the Netherlands

^12^ Aberdeen Health Psychology Group, Institute of Applied Health Sciences, University of Aberdeen, Aberdeen, UK.

^13^ Department of viro-science, Erasmus Medical Center Rotterdam, the Netherlands

^14^ Department of Internal Medicine and Infectious Diseases, Erasmus University Medical Center, Rotterdam, the Netherlands

^16^ Department of Infectious Diseases, Leiden University Medical Centre, Leiden, the Netherlands

^17^ Center of Infection and Immunity Amsterdam (CINIMA), Department of Neurology, Academic Medical Center, Amsterdam, The Netherlands

^18^ Laboratory of Experimental Immunology, Academic Medical Center Amsterdam, the Netherlands

^19^ Laboratory for Viral Immune Pathogenesis, Academic Medical Center Amsterdam, the Netherlands

^20^ Department of Pharmacy, Radboud University Nijmegen Medical Center, the Netherlands

^21^ Immunology Laboratory, Vaccine Research Center, NIAID, National Institutes of Health

^22^ US Military HIV Research Program and the Henry M. Jackson Foundation for the Advancement of Military Medicine, Bethesda, United States

^23^ Department of Virology, Erasmus Medical Center, Rotterdam, the Netherlands

^24^ Department of Internal Medicine, Free University Medical Center, Amsterdam, the Netherlands

^25^ Department of Internal Medicine, Slotervaart Hospital Amsterdam, the Netherlands

^26^ DC Clinics, Amsterdam, the Netherlands

^27^ Department of Internal Medicine, Lukas Andreas Hospital, Amsterdam, the Netherlands

^28^ School of Public Health, Faculty of Medicine, Imperial College London, London, United Kingdom

^29^ Department of Internal Medicine, Radboud University Nijmegen Medical Center, the Netherlands

^30^ Health Centre Venserpolder, Amsterdam, the Netherlands

^31^ Sexology Centre Amsterdam, the Netherlands
